# The isolation and identification of *Bacillus velezensis* ZN-S10 from vanilla (*V. planifolia*), and the microbial distribution after the curing process

**DOI:** 10.1038/s41598-024-66753-z

**Published:** 2024-07-16

**Authors:** Thabani Sydney Manyatsi, Yu-Hsin Lin, Ying-Tzy Jou

**Affiliations:** 1 Department of Tropical Agriculture and International Cooperation, National Pingtung University of Science and Technology, Neipu Shuefu Road 1, 91201 Pingtung, Taiwan; 2 Department of Biological Science and Technology, National Pingtung University of Science and Technology, Neipu Shuefu Road 1, 91201 Pingtung, Taiwan

**Keywords:** Vanilla, Curing process, 16S rRNA gene sequencing, Bacterial fermentation, *Bacillus velezensis* ZN-S10, Biological techniques, Isolation, separation and purification, Microbiology techniques, Sequencing, Bacteria, Microbial communities

## Abstract

The market value of vanilla beans (*Vanilla planifolia*) is constantly increasing due to their natural aroma and flavor properties that improve after a curing process, where bacteria colonization plays a critical role. However, a few publications suggest that bacteria play a role in the curing process. Hence, this study aimed to isolate *Bacillus* sp. that could be used for fermenting *V. planifolia* while analyzing their role in the curing process. *Bacillus velezensis* ZN-S10 identified with 16S rRNA sequencing was isolated from conventionally cured *V. planifolia* beans. A bacteria culture solution of *B. velezensis* ZN-S10 (1 mL of 1 × 10^7^ CFU mL^−1^) was then coated on 1 kg of non-cured vanilla pods that was found to ferment and colonize vanilla. PCA results revealed distinguished bacterial communities of fermented vanilla and the control group, suggesting colonization of vanilla. Phylogenetic analysis showed that ZN-S10 was the dominant *Bacillus* genus member and narrowly correlated to *B. velezensis* EM-1 and *B. velezensis* PMC206-1, with 78% and 73% similarity, respectively. The bacterial taxonomic profiling of cured *V. planifolia* had a significant relative abundance of *Firmicutes, Proteobacteria, Cyanobacteria, Planctomycetes,* and *Bacteroidetes* phyla according to the predominance. *Firmicutes* accounted for 55% of the total bacterial sequences, suggesting their colonization and effective fermentation roles in curing vanilla.

## Introduction

Vanilla (*Vanilla planifolia*) is a tropical orchid (Orchidaceae family) known worldwide for its aroma or flavor properties^1,2^. In *V. planifolia*, more than 200 compounds contribute to its distinctive flavor and aroma, with vanillin being the most abundant component^1,3^. Hence commercially, vanillin is required in vast quantities. Unfortunately, vanilla plants cannot supply adequate vanillin for the global market^4^. Flavoring agents such as vanillin (3-methoxy, 4-hydroxy-benzaldehyde), anise alcohol, guaiacol, and *p*‐hydroxybenzaldehyde, found in natural cured vanilla are widely used in food or baking products, cosmetics, aromatherapy, and pharmaceuticals for various purposes^5,6^. Thus, vanilla beans can provide natural vanillin but are limited and costly^7,8^. To obtain a natural vanilla extract, *V. planifolia* must undergo a laborious and protracted curing procedure that also improves the vanilla's aroma^9,10^.

In an agricultural farm or a greenhouse environment, matured green vanilla beans lack the distinctive vanilla flavor and are harvested odorless^11,12^. Hereafter, at post-harvest, a curing process is performed to initiate the formation of vanilla beans' distinctive aroma, flavor, and color properties. The conventional curing technique involves blanching or killing, fermentation (sweating), slow-drying, and conditioning the vanilla beans^13–15^. According to Peña-Barrientos et al.^16^, the curing process alters the physicochemical, and structural properties of vanilla beans, increasing the aromatic compounds of the bean, while the flavor attribute is also developed. Notably, fermented vanilla beans and their products have been reported to have an added distinctive aromatic value^17^. Other researchers have used the *Bacillus*-curing process to form better aromatic and flavor attributes caused by the hydrolysis of glucovanillin and the formation of vanillin compounds^18^. Hence, these studies have exhibited that *V. planifolia* can be colonized by *Bacillus* isolates inoculated on vanilla bean pods. The glucosidase produced by these isolates influences flavor production by hydrolyzing glucovanillin. Thus, the colonization of *Bacillus* in vanilla is tangled in flavor and aroma development during fermentation or the “sweating” stage in the curing process^19^.

Fermentation in *V. planifolia* is anticipated as a strategy or valorization technique to obtain natural vanillin from vanilla beans, wherein glucovanillin is converted to vanillin. Hence, *Bacillus* sp., lactic acid bacteria (LAB), and other studied bacteria are added to green-uncured vanilla beans to improve their volatile profile^17,20^. It should also be noted that during fermentation, glucovanillin is hydrolyzed by glucosidase produced by *Bacillus* colonies on vanilla bean pods^21^. Buitimea-Cantua et al.^22^ reported that during the curing process of *V. planifolia*, the key precursors of vanillin (β-d-glucovanillin and vanillyl alcohol) are augmented during the sweating owing to the enzyme β-d-glucosidase activity that is influenced by pressure and moisture during this treatment. Similarly, vanillin is increased hence improving the flavor or aroma development and enzymatic activity^23^. Vanilla's glucovanillin content increases with the browning of the vanilla pods; after fermentation, the beans tend to have a higher glucose and vanillin content, subsequently an increased market value is attained^24–26^. The colonization of microorganisms on vanilla bean (*V. planifolia*) plays an essential role in flavor and aroma formation^18,21^. Gu et al.^19^ mentioned that the number of microorganisms such as bacteria communities that colonize *V. planifolia* during plant growth, at harvest, and after the curing process is numerous. In this study, the indirect aromatic and flavor profile influence of vanilla by microorganisms was explored with an investigation of the bacterial communities involved during the fermentation or sweating stage of the curing process.

Studies have shown that a 16S rRNA gene sequencing method analyzes bacteria and other microorganisms in a specific environmental sample to study the composition of the microbial population, and deduce the diversity, and abundance while exploring the relationship between microorganisms and the treatment^27–29^, hence it has been also observed by Roling et al.^30^ in a vanilla flavor development study. Next-generation sequencing (NGS) technologies based on 16S rRNA gene sequencing are evolving rapidly, and it has become possible to sequence the entire genomes or transcriptomes of agricultural produce efficiently and economically^31–33^. Moreover, De Vrieze et al.^34^ mentioned that 16S ribosomal RNA-based analysis could be established as an accepted technique for profiling bacterial or fungi communities.

The research anticipated that the application of bacterial inoculates on vanilla might influence the fermentation of vanilla pods with edible microorganisms, rather than allowing the curing of vanilla traditionally which might include contaminants. Notably, few reports exhibited the involvement of bacteria communities while presenting the taxonomy and related profiling of these microorganisms. Therefore, this study isolated *Bacillus velezensis* ZN-S10 strain from vanilla beans, to objectively employ bacterial fermentation of the obtained isolate (ZN-S10) in non-cured vanilla pods to explore the vanilla microbiome that may be associated with the curing process during the fermentation stage of *V. planifolia*. The phylogeny and taxonomy of the vanilla microbiome focused on bacteria communities involved when *V. planifolia* pods were fermented with *B. velezensis* ZN-S10 compared with traditionally cured vanilla beans was established.

## Materials and methods

### Sample preparation

Green, matured vanilla (*V. planifolia*) bean samples were collected from a local farm in Pingtung, Taiwan (22°25′41.8″N 120°32′29.0″E). A 1 kg vanilla bean pod pack was washed with sterilized water and then blanched in hot water (~ 80 °C) for 3 min. The blanched vanilla pods were instantly placed in cooling boxes with paper tissues to let the pods dry out. According to Xu et al.^18^, this “killing” stage inhibits the vegetative growth of the beans while allowing enzyme activation and initiating color change (from yellow/green to dark brown) and the flavor or aroma development of the final cured vanilla bean.

### Curing of vanilla bean pods

The blanched vanilla beans were allowed to naturally ferment in a cool freezer; wherein the samples were stored in plastic boxes and wrapped in tissue paper and stored at 10 ℃. It should be noted that the tissue paper was changed every 2 days to avoid mold formation. Occasionally, cool-air drying and humidification were also done once a week with a lamina flow and a humidifier, respectively. Additionally, as the beans dried and showed yellow to brown color changes owing to phenol browning and lipid oxidation that occur simultaneously with the enzymatic hydrolysis of vanillin precursors^22^. Thus, brown to dark brown vanilla bean pods without molds were recognized as the best-quality cured samples and were used for experimentation.

### Bacteria isolation and purification

A batch of the brown-dry cured vanilla bean pods was cut into 0.3–0.5 cm pieces and ground into 0.5 g powder. Sterile distilled water was then added in 15 mL tubes with the vanilla powder, then 20 min agitation in ultrasound (Ultrasonic Bath Delta Model DC 150-H, Takashi, Japan) and stored at 20 ℃ overnight, following the method of Chen et al.^28^. A de Man, Rogosa, and Sharpe (MRS) agar medium^35^ were modified and prepared on Petri dishes, maintained at pH 5.7, and autoclaved for 15 min. The altered and selective MRS media was composed of dextrose of 15 g L^−1^, yeast extract (5 g L^−1^), 5 g L^−1^ of sodium acetate, potassium phosphate of 2 g L^−1^, magnesium sulfate of 0.1 g L^−1^, and 1.00 mL drop of Tween 80. A supernatant of 100 µL was inoculated on MRS agar and then spread on the Petri dishes for oven incubation at 37 ℃ for 16 h. After the incubation, the bacteria were selected after three consecutive plates according to shape, color, and size to obtain pure isolates. The re-streaking method on the MRS agar was used as a purification technique for the bacteria colonies. Hence, the pure isolates were selected for identification and were stored in 20% glycerol at − 80 ℃ for further experiments.

### Hemolysis test

The blood agar test was conducted with pure collected bacterial isolates to observe the safety of using these microbes for the food industry and animal use. The safety assessment was based hemolytic reaction recognized by lyses of blood cells on the blood agar^36^, hence the isolated bacteria colonies were tested with the hemolysis assay. In 1 L of distilled water, 40 g of a commercial agar-based medium (Infusion agar) (Himedia Laboratories Co., India), was suspended and then heated to boil at 121 ℃ at 15 lbs pressure. The agar medium was then allowed to cool and 5% *v/v* of sterile sheep-defibrinated blood was added and mixed well before pouring into Petri plates. The pure isolate bacteria were then streaked on these blood agar plates and then incubated at 37 ℃ for 16 h to observe the alpha, beta, and gamma properties of the bacteria. According to studies; *Staphylococcus* sp., *Streptococcus* sp., and *Enterococcus* sp. can be differentiated by the degree of hemolysis caused by their hemolysis^37,38^. Hence, the bacteria with beta (β) hemolysis^39^ had been classified as complete hemolysis seen with the formation of a clear-transparent zone, and the plate with those properties was discarded in this study. Partial hemolysis was recognized with opaque-green zone formation on the blood agar plates, thus was also discarded in this study. The agar plates with notable growth on the blood agar medium characterized as gamma (γ) hemolysis were the targeted bacteria colonies and were used for identification and subsequent experiments of interest in this research. However, the bacteria with alpha (α) hemolysis were observed with partial hemolysis showing rupture or “digestion” with a lightened (yellow) and transparent area on the blood agar medium were discarded. Hence, based on the above-mentioned literature, this study performed a visual assessment of the hemolysis (α, β, γ) for food safety purposes.

### Identification of the bacteria

A 16S rRNA gene sequencing technique^40^ was used for the identification and classification of the pure bacteria isolates. The 16S rRNA molecular sequencing identification was performed by Mission Biotech Company (Taiwan), and arranged with ABI Big Dye Terminator v3.1. PCR mixtures were amplified by initially holding at 94 °C for 5 min. Besides, 40 cycles of denaturing at 94 °C for 30 s, annealing at 55 °C for 30 s, and extensions at 72 °C for 2 min 20 s. Then the reaction ended with a final extension at 72 °C for 5 min and a hold at 4 °C. In this study, the amplification of the 16S rRNA was F8 (5ʹ-AGAGTTTGATCMTGGCTCAG-3ʹ) and R1510 (5ʹ-CGGTTACCTTGTTACGACTT-3ʹ)^41^ primers, respectively. It should also be noted that the nucleotide sequences are obtainable on the National Center for Biotechnological Information (NCBI) website where the Basic Local Alignment Search Tool (BLAST) was used for phylogenetic analysis.

### Phylogenetic tree analysis based on 16S rRNA gene sequences

The evolutionary data of the phylogenetic tree was analyzed using the Neighbor-Joining method^42,43^. With branch lengths in the equal units as those of the evolutionary distances used to deduce the phylogenetic tree, the ideal tree was drawn to scale (2.00). The evolutionary distances are expressed in base substitutions per site and were calculated using the Maximum Composite Likelihood approach^44^. Beside each internal node in the tree is the percentage of sites where at least 1 unambiguous base is present in at least 1 sequence for each descendent clade is shown next to each internal node in the tree. This analysis involved 10 nucleotide sequences. All ambiguous positions were removed for each sequence pair (pairwise deletion option). There was a total of 96 positions in the final dataset. Evolutionary analyses were conducted and extracted NCBI 16S rDNA sequences from the aforementioned genome sequences and aligned them using MEGA11^45^. The sequences were subjected prior to the NCBI database (https://www.ncbi.nlm.nih.gov/) for a FASTA search as well as obtaining the accession numbers according to the sequences.

### Fermentation of *V. planifolia*

A new batch of green matured vanilla bean pods (non-blanched) was sorted according to sizes of approximately 15 cm, considered the best quality. The pods (1 kg) were blanched for 2 min in hot water (~ 80 ℃) and then dried for 3 h. After the quick drying, the 1 kg bean pods were placed in a plastic box. Then liquid *Bacillus velezensis* ZN-S10 bacteria culture of 1 mL of 1 × 10^7^ CFU mL^−1^ was sprayed on the vanilla pods and the plastic box was shaken to allow the bacteria to colonize and ferment the vanilla pods. The UV–Vis spectrometer (DU 640, Micro-Digital Co., Korea) was used to measure the OD value _600_ of the liquid to obtain the desired concentrate for bacterial fermentation. These bacteria-fermented samples were considered as the treatment group, while the vanilla beans that were uncured and allowed to ferment naturally i.e. without the addition of any isolated bacteria; were categorized as the control group.

### Bacteria community analysis

The purified bacteria isolates were isolated for DNA extraction using a Qiagen DNeasy Plant Kit (TopGen Biotechnology Co., Ltd, Kaohsiung, Taiwan). The DNA sequencing model was an ABI 3730XL DNA Analyzer using the primers 907R and 16sF-1F with an ABI Big Dye Terminator v3.1 reagent. The sample and 400 µL API buffer were added and heated to 65 ℃ for 15 min. This procedure was repeated twice for 8 min. The tubes were placed on ice for 5 min. The samples were centrifuged at 14,000 rpm, at 25 ℃ for 5 min. The supernatant was discarded and the lower solution was resuspended with AW1 buffer (1.5 times the volume of supernatant). At this step, a min centrifuge at 8000 rpm was done at room temperature. 50 µL of 40 ℃ warm water was added and allowed to settle for 10 min. This tube was then subjected to centrifugation at 14,000 rpm for 1 min.

### Taxonomic profiling and Venn diagram construction

The microbial communities for each unique sample group were characterized according to their taxonomic profile using the relative microbial abundance. Only the bacterial OTUs of the phylum and genus levels were used for analysis in this research. Hence, to compare the genus and phylum levels between the bacterial communities for non-cured vanilla beans and bacteria-fermented (cured) samples were presented in a Venn diagram (in OTUs).

### Principal component analysis (PCA)

Statistical analysis or chemo-metrics can be employed after the curing process to observe the vanilla microbiome. A multivariate statistical approach; principal component analysis (PCA) can be used to minimize the dimensionality of a set of observations made up of a lot of interconnected variables^46,47^. The PCA was used in this study as a technique for analyzing the bacterial microbial communities. This technique is an ordination of reduced variables that are projected at the initial set of variables into new axes (PCs). Hence, the first-hand axis (PC1) is calculated on another axis (PC2) as an eigenvalue of the correlation (or covariance) matrix which is attained from the multivariate matrix of the data set, so a restricted number of axes extracts the highest possible variance, and eigenvectors linked with eigenvalues that decide each PC direction^48,49^.

### Taxonomic abundance profiling of the bacterial communities

According to the abundance of information on the bacterial communities at the taxonomic level, a histogram at the phylum level and a heat map analysis at the genus level based on the Bray–Curtis distance were constructed to calculate the frequency of related communities of non-treated vanilla samples and bacteria fermented group. The grouping of the related bacterial communities that may influence the curing process during the fermentation of *V. planifolia* was exhibited in a bubble plot and a circos presentation at phylum and genus levels, respectively.

### Ethical statement

The authors would like to declare that vanilla bean pods (*Vanilla planifollia*) were harvested, green and matured with permission from Pingtung Lin Tsai Bian Association Farm, Taiwan (22°25′41.8″N, 120°32′29.0″E). The website link of the farm is as follows https://www.agriharvest.tw/archives/73970 confirming that the harvest of the vanilla was grown under the appropriate shade and cultivation media before it was harvested and used in this study. The farm also confirmed that the vanilla was following the national regulations of the Ministry of Agriculture in Taiwan (https://eng.moa.gov.tw/); hence vanilla bean pods were grown following international guidelines. Authors do not have any conflict of interest.

## Results and discussion

### Strain identification

The bacteria used in this study was isolated from vanilla beans (*Vanilla planifolia*). The isolation was done from previously naturally fermented and cured batches of vanilla bean pods in the laboratory. A 16S rRNA gene sequencing was conducted based on Johnson et al.^50^ methods owing to high accuracy compared to other molecular identification markers. For instance, at the species level of *Acinetobacter* identification 98.2% accuracy was reported with 16S rRNA gene sequencing, *rpoB* gene sequencing had 93.4%, while 77.2% was with *gyrB* multiplex PCR, and the VITEK 2 technique had 35.9% accuracy^51^. Mohkam et al.^52^ noted that discerning *Bacillus* isolates with *rpoB* gene sequencing was sometimes unsuccessful. In this study, the isolated bacteria strain was *Bacillus velezensis* ZN-S10 acquiring a complete genome. The *Bacillus* genus is classified as a gram-positive bacterium, from the *Bacillaceae* family. It is rod-shaped and aerobic with the formation of endospores; hence, it showed whitish, round, and medium-sized colonies in this study, as reported in other research^53–55^.

### Characterization of isolated pure colonies and hemolysis test findings

**Figure 1:**
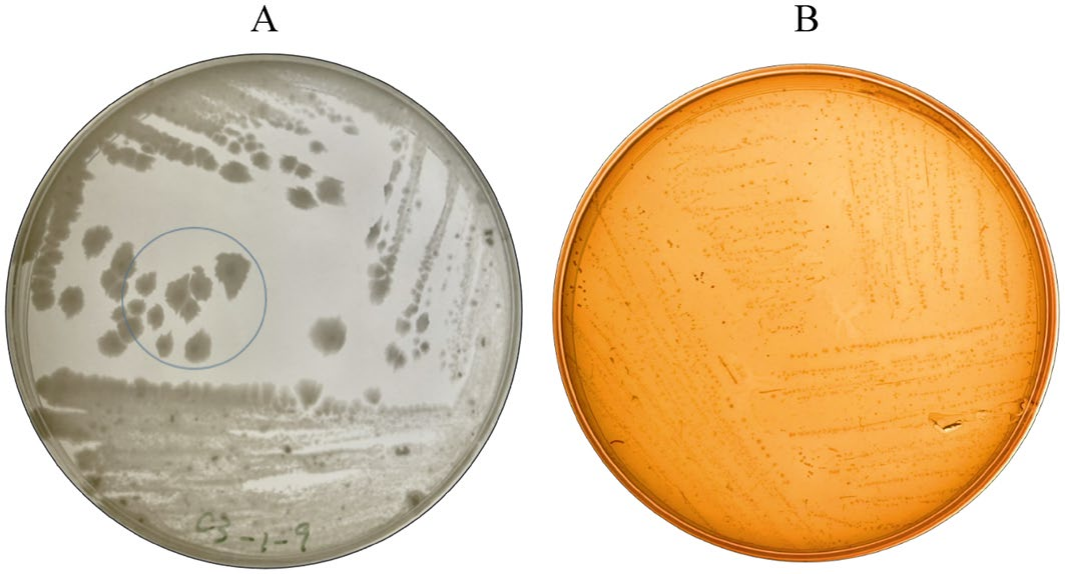
*Bacillus velezensis* ZN-S10 colonies. The ZN-S10 pure isolate appearance showing the single colonies on the fourth streak region (circled) grown on MRS agar Petri-plate, incubated at 37 ℃ for 16 h (**A**); and the gamma hemolysis of the ZN-S10 strain (**B**) grown on blood agar.

This study found that *Bacillus velezensis* ZN-S10 colonies were effectively isolated from vanilla bean pods (*V. planifolia*) when grown with an overnight culture in MRS agar plates and incubated at 37 ℃ for 16 h. As observed in Fig. 1, the colonies were fairly spread with off-white colonies on the surface of MRS agar Petri dishes after three consecutive plates (MRS subculture), hence the single pure colonies (Fig. 1A) were found. Researchers in other studies have reported that the MRS medium can be used as a selective medium supporting the growth of *Lactobacilli* and *Bacillus* bacteria strains, due to the vitamins, carbon, and nitrogen found in sodium acetate and ammonium citrate of the MRS composition^56,57^. Hereafter, miscellaneous microorganisms, especially bacteria in the environment are eliminated while aiming for specific edible bacteria for vanilla food processing.

The hemolysis test characterized by Savardi et al.^58^ as alpha, beta, and hemolysis was conducted with blood agar. The isolated bacteria (*B. velezensis* ZN-S10) exhibited gamma hemolysis (Fig. 1B) exhibiting no hemolysis since the bacteria did not absorb blood agar. The absence of hemolysis showed that the pure isolate was safe for vanilla fermentation use in both handling, and application, hence ZN-S10 can also be edible for human consumption.

According to this study, 11 strains were isolated from traditionally cured vanilla bean pods. A 16S rRNA gene sequencing was done and identified more than 98.5% partial genomes of 10 *Bacillus* species. *Bacillus velezensis* ZN-S10 was the only strain with a complete genome sequence, at 100% identity, thus it was selected as the appropriate strain to investigate the capability of bacterial strains in the effective colonization and fermentation of vanilla bean pods. The accession numbers of the isolated bacteria strain from *V. planifolia* used in this study, can be obtained from the NCBI website (https://www.ncbi.nlm.nih.gov/) for the nucleotide sequences. The accession number and sequence length (in bp) of the ZN-S10 strain have been presented in the Supplementary Section. These gram-positive bacteria were effectively isolated from *V. planifolia*. Similarly, Escobar-Mucino et al.^59^ isolated and identified microbial species of *Pseudomonas* sp., *Citrobacter* sp., and *Enterobacter* sp. with 16S rRNA gene sequencing, from Mexican *Vanilla planifolia* ex. Andrews. Moreover, in our previous study, vanilla beans treated with *B. velezensis* ZN-S10 showed the highest vanillin content of 2.59% when compared to 0.38% vanillin on non-coated vanilla. Studies have shown that the vanillin content of high-quality vanilla ranges between 1.7 and 3.6% for *V. planifolia* and 1.0–2.0% for *V. tahitensis*^30,60,61^ owing to the indirect influence of microorganisms. Hence, the bacterial genome of *Bacillus velezensis* ZN-S10 with the potential of increasing vanillin in *V. planifolia* was investigated in this study.

### The phylogenetic tree of the evolutionary relationship of taxa

**Figure 2:**
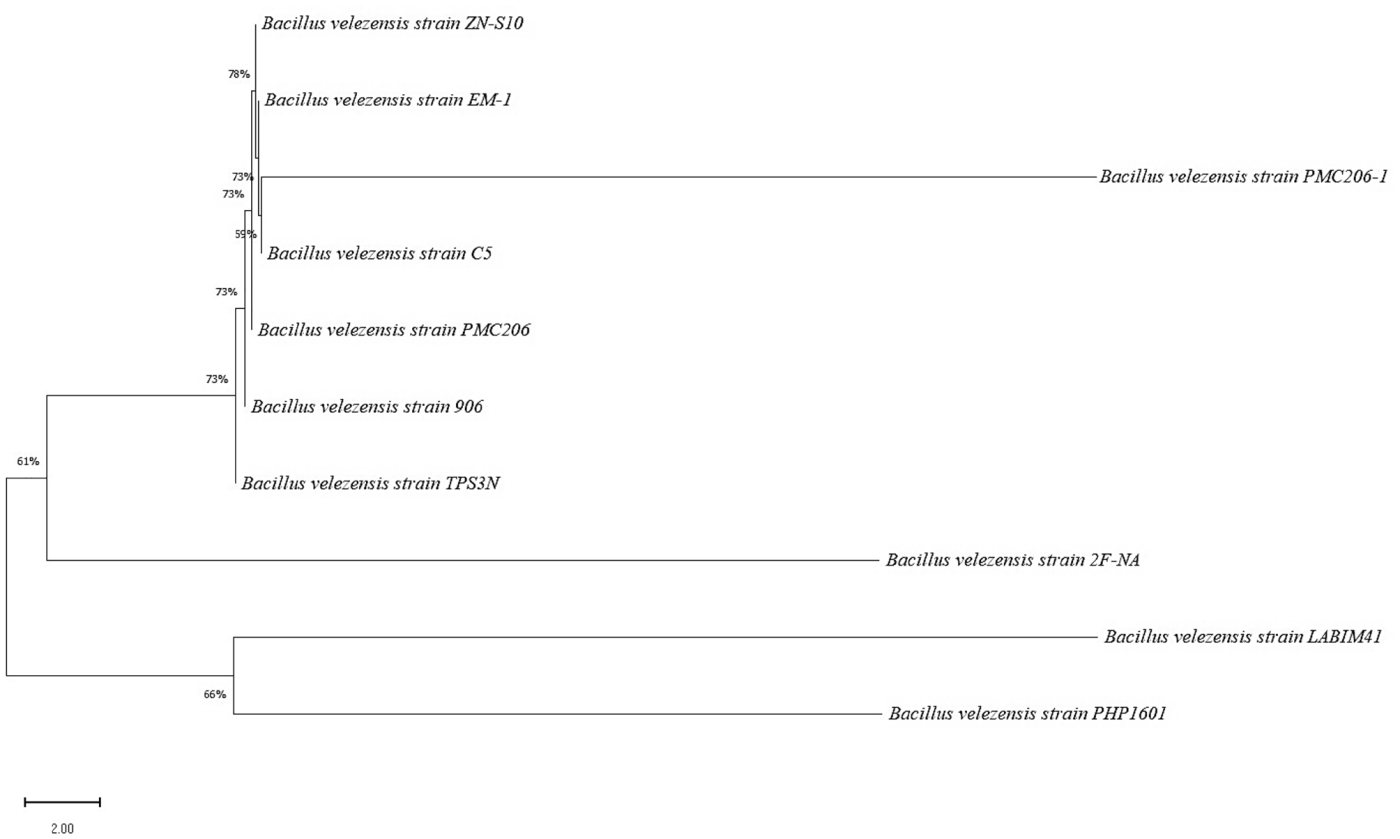
The phylogenic tree of 10 random sequences from the *Bacillus velezensis* strains, highlighting their position within the *Bacillus* genus. The tree was built with MEGA, using the neighbor-joining technique. The percentage numbers at nodes are of bootstrap values, calculated by 1000 repeats of the tree. This analysis involved 10 nucleotide sequences of *Bacillus velezensis* strains, with a total of 96 positions in the final dataset.

In this study, the phylogenetic tree was constructed based on the 16S rRNA gene sequences, where the accession numbers of the isolated strain and related sequences were obtained from the NCBI web database (https://www.ncbi.nlm.nih.gov/). Bacterial phylogenetic analyses are frequently carried out to investigate the evolutionary relationships among distinct bacterial species and genera^62^. Hence, we performed the comparison of the *Bacillus velezensi*s strains acquired from non-cured vanilla beans employing the Neighbor-Joining method to show the phylogenetic relationships of the strains (Fig. 2). Yeh et al.^6^ reported that microbial coating with *Bacillus subtilis* subsp. *subtilis* on *Vanilla planifolia* yielded fermentation products and volatile components that contributed better aromatic profile of vanilla. Hence, with future studies, the *B. velezensi*s strain ZN-S10 used for fermentation was found distinct from other members of the *Bacillus* group which might alter the aromatic characteristics of vanilla. Hence, based on the constructed phylogenetic tree (Fig. 2), *B. velezensis* ZN-S10 were the major bacteria that colonized the vanilla bean pods, and the strain was used for fermentation at the curing process for a new batch of non-cured vanilla bean pods. The strain ZN-S10 accounted for the bootstrap value of 79%, with lesser differences of similar isolate sequences as neighbor strains of *B. velezensis* EM-1, PMC206-1, and C5 strains with bootstrap values of 73%. The *Bacillus* genus exhibited that the colonizing microbes were involved the coating of the vanilla pods with ZN-S10 strain, based on the 16S rRNA as illustrated by the phylogenetic tree. Other studies have shown that during the curing of *Vanilla planifolia* Andrews, *Bacillus* isolates (such as *Bacillus vanillea* XY18, *B. subtilis* XY11, and *B. subtilis* XY20), can colonize the vanilla beans^19,21^.

### Comparison and variation of bacterial communities in naturally fermented and treated vanilla

**Figure 3:**
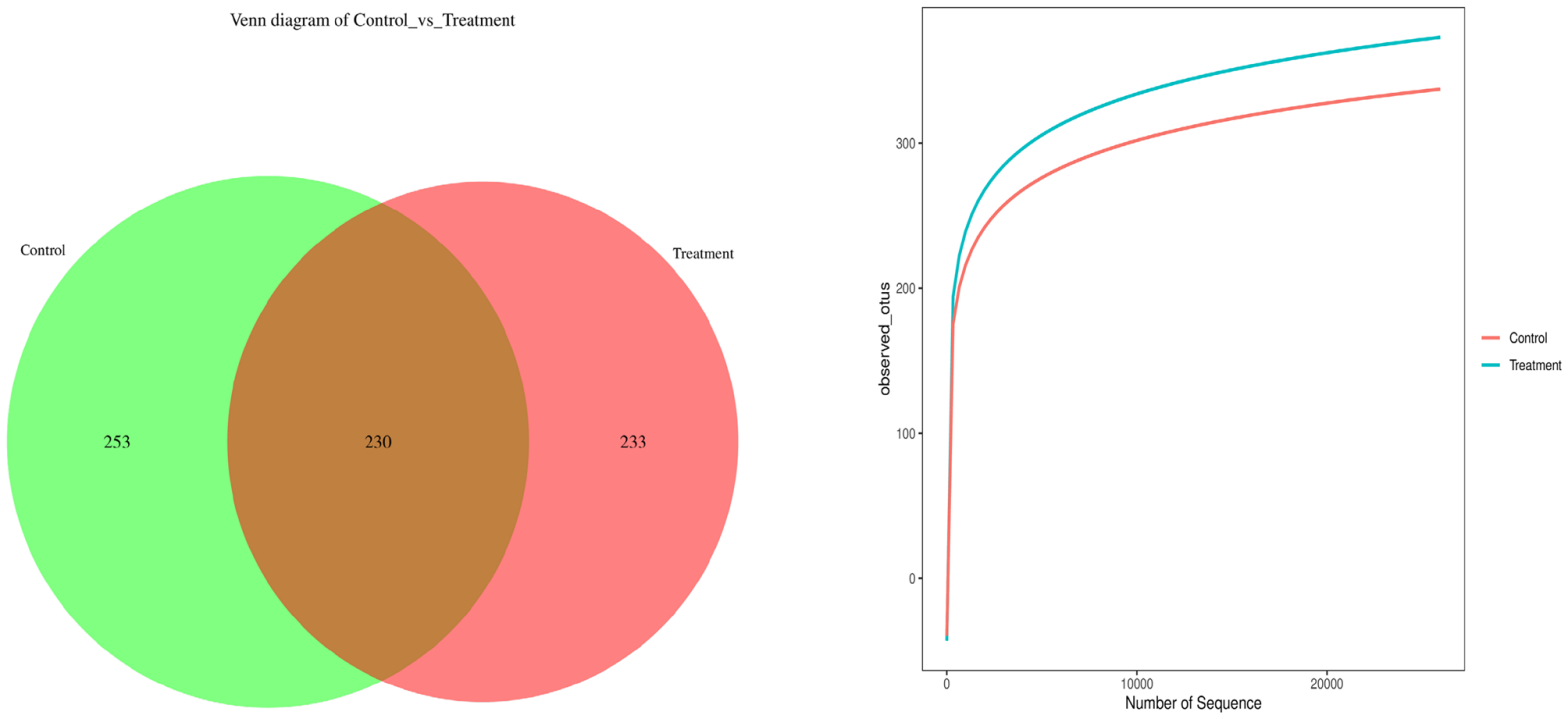
Venn diagram (left) at shared and the unique OTUS of the vanilla microbiome and schematic diagram (right) of dilution curve results of naturally fermented (control) vanilla beans and *Bacillus velezensis* ZN-S10-treated samples. representing the control vanilla beans and *Bacillus velezensis* ZN-S10-treated samples.

A Venn diagram (Fig. 3) was constructed for the number of common (uncured vanilla beans) and unique species (bacteria-fermented cured vanilla) in each group. All the sequences of each sample were compared with the NCBI RefSeq 16S rRNA database using EPI2ME v3.0 (Oxford Nanopore Technologies, UK). Hence, the number of variants was very similar to the mean expression of species in the mean groups; showing 230 OTUs amongst the naturally fermented vanilla beans and a very similar population of 233 OTUs in the treatment group. Between the two groups (control and treated vanilla), 230 OTUs were shared by both, while the non-treated pods had the majority of 253 OTUs in the bacterial communities. The shared OTUs between the naturally fermented vanilla (control) and *B. velezensis* ZN-S10 fermented samples suggested the successful colonization with microorganisms; hence effective fermented and cured vanilla beans. Similarly, Chen et al.^21^ reported that *Bacillus* sp. colonizes vanilla beans and is classified as β-d-glucosidase-producing bacteria. The bacterial community population was analyzed with 16 s rRNA gene sequencing presented in operational taxonomic units (OTUs) as shown in Fig. 3 (right). The results reported that the bacteria-fermented vanilla beans had a higher population (~ 390 OTUs) than the control as expected; hence showing the success of curing the beans with *B. velezensis* ZN-S10 used as a fermentation tool. Studies have also proved that bacteria communities are involved during the curing process of vanilla beans^18^. Hence, the occurrence of the ZN-S10 showed their role in the effective fermentation of *V. planifolia* during the curing process.

### Principal component analysis of the bacterial communities

**Figure 4:**
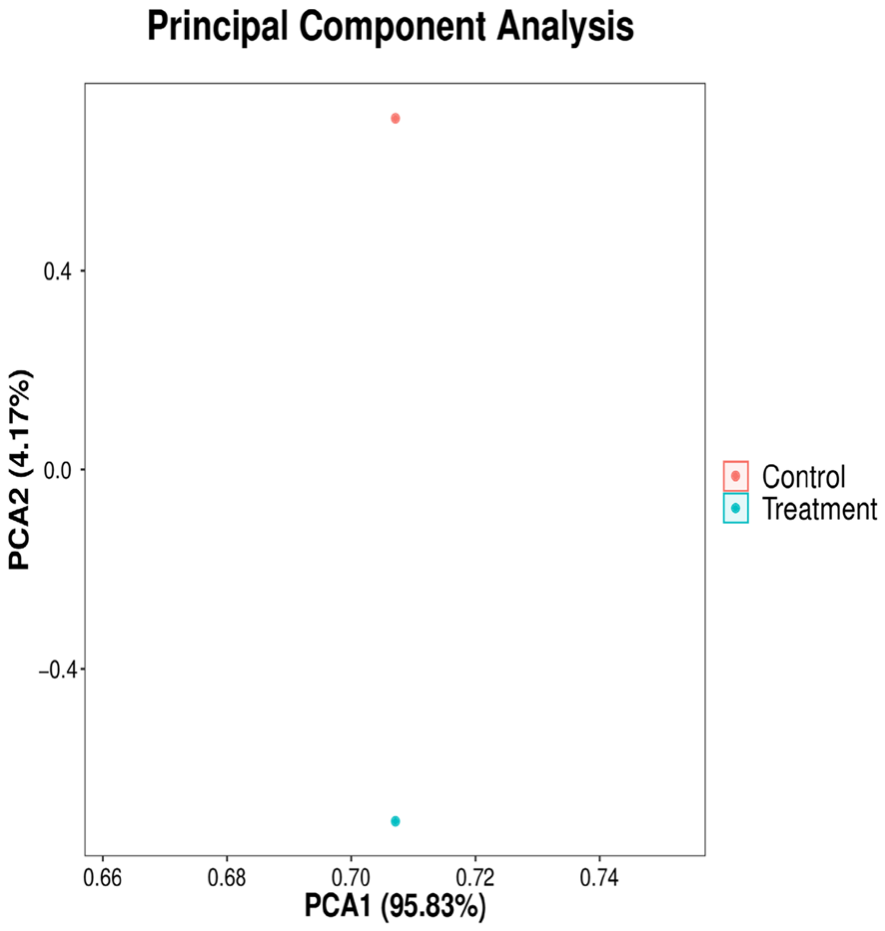
The principal component analysis (PCA) of the bacterial communities obtained from naturally fermented vanilla (control) and *B. velezensis* ZN-S10-treated vanilla beans.

The microbial communities were profiled with principal component analysis (PCA) to visualize the differences between microorganisms obtained from naturally fermented vanilla and the beans that were treated with 1 mL of 1 × 10^7^ CFU mL^−1^ of *B. velezensis* ZN-S10*.* As presented in Fig. 4, the microbial population accounted for 95.83% of PC 1 of the total variance in treated samples, while control samples showed that PC 2 accounted for 4.17% in the PCA score plot. The presence of endophytic bacteria has been shown on non-bacteria cured vanilla pods (control) and *B. velezensis* ZN-S10 treated samples, as also found in other studies. For instance, a root-colonizing *Bacillus amyloliquefaciens* isolated by White et al.^63^, was also isolated in leaves, flowers, and pods of *Vanilla planifolia*. Likewise, researchers have reported that Colombian vanilla had a couple of endophytes on the plant even on harvested pods^64^.

### Findings on the microbiome of the vanilla before and after fermentation with *B. velezensis*

**Figure 5:**
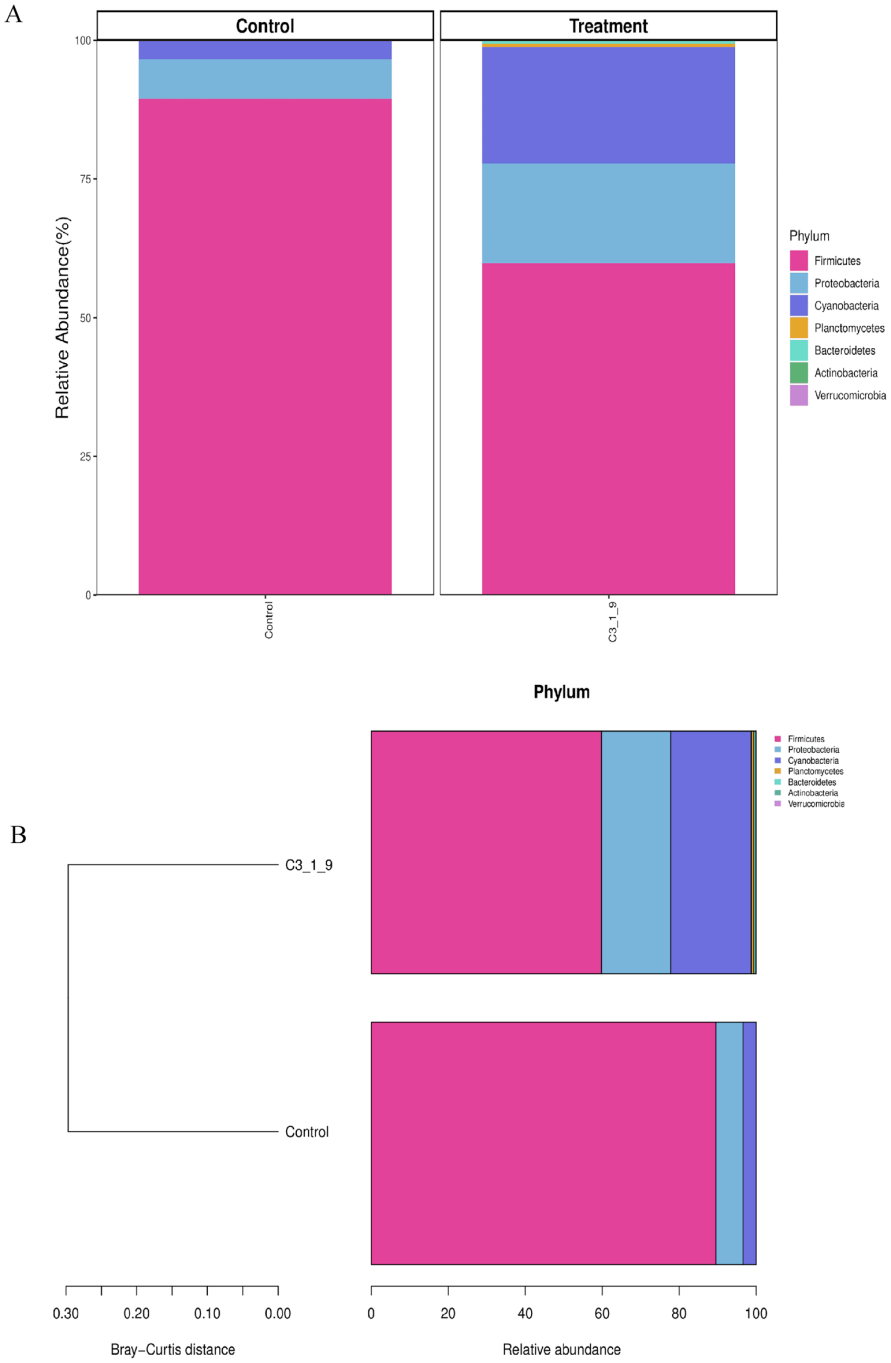
The bacterial abundance of the vanilla microbiome. (**A**) Histogram of the bacterial composition at its relative abundance (%) presented at the phylum level of the control group (naturally fermented vanilla pods) and *Bacillus velezensis* ZN-S10 fermented vanilla (C3-1-9). (**B**) The relative sample abundance at the phylum level was analyzed with the Bray–Curtis distance of non-treated vanilla samples (control) and *B. velezensis* ZN-S10 fermented (C3-1-9).

The vanilla microbiome after fermentation with *B. velezensis* ZN-S10 was evaluated with high-throughput amplicon sequencing of the bacterial 16S rRNA gene showed a predominance of *Firmicutes, Proteobacteria, Cyanobacteria, Planctomycetes,* and *Bacteroidetes*, respectively (Fig. 5). These genera made up ∼95% of the total data set wherein at phylum level, a high dominance (≈ 80%) of *Firmicutes* with the subdominant of *Proteobacteria*, and a small fold of *Cyanobacteria* was observed (Fig. 5A) with the vanilla beans that were blanched and allowed to ferment naturally. Nevertheless, the vanilla samples fermented with isolated bacteria from previously naturally fermented vanilla batches also showed a high dominance of *Firmicutes,* (≈ 60%) which was slightly lower in abundance with control samples. The microbiome of treated samples was followed by *Proteobacteria* and *Cyanobacteria* which were a two-fold increase as compared to control samples (Fig. 5A). Researchers have also reported that *Proteobacteria* and *Cyanobacteria* were the common families found in the core of the vanilla microbiome^27^. Also, according to Xiong et al.^65^, the monoculture practice of growing vanilla beans contributes to the high dominance of *Firmicutes* due to low bacterial and fungal communities that occur during the continuous cropping of the vanilla beans. Furthermore, the relative abundances of *Firmicutes, Actinobacteria, Bacteroidetes*, and *Basidiomycota* are common phyla in vanilla plants especially when grown in a monoculture system. Hence, in this study, the results indicated that at a phylum level *Firmicutes, Proteobacteria,* and *Cyanobacteria* were the bacterial groups (Fig. 5A and B) with key influence in the curing of vanilla beans. The treated vanilla bean pods showed a higher relative abundance of *Cyanobacteria* and *Proteobacteria* as compared to the non-treated samples (Fig. 5B). Moreover, it should be noted *Planctomycetes*, and *Actinobacteria* were presented in the bacteria-fermented vanilla although the communities in this group were in small abundance, rather than in the control samples (Fig. 5B). Notably, the discussed findings of the bacteria communities were found on the cured *V. planifolia* beans for both treated samples (ZN-S10 fermented) and non-treated pods. The availability of the *Bacillus* sp. might contribute to the aroma and flavor properties of the vanilla pods. Gu et al.^19^ found that cured pods with *B. vanillea* and *B. subtilis* coating had elevated vanillin yielding better aromatic qualities of vanilla. However, it should be noted that different geographic locations of vanilla species vary in flavor and aroma attributes^26,66^.

**Figure 6:**
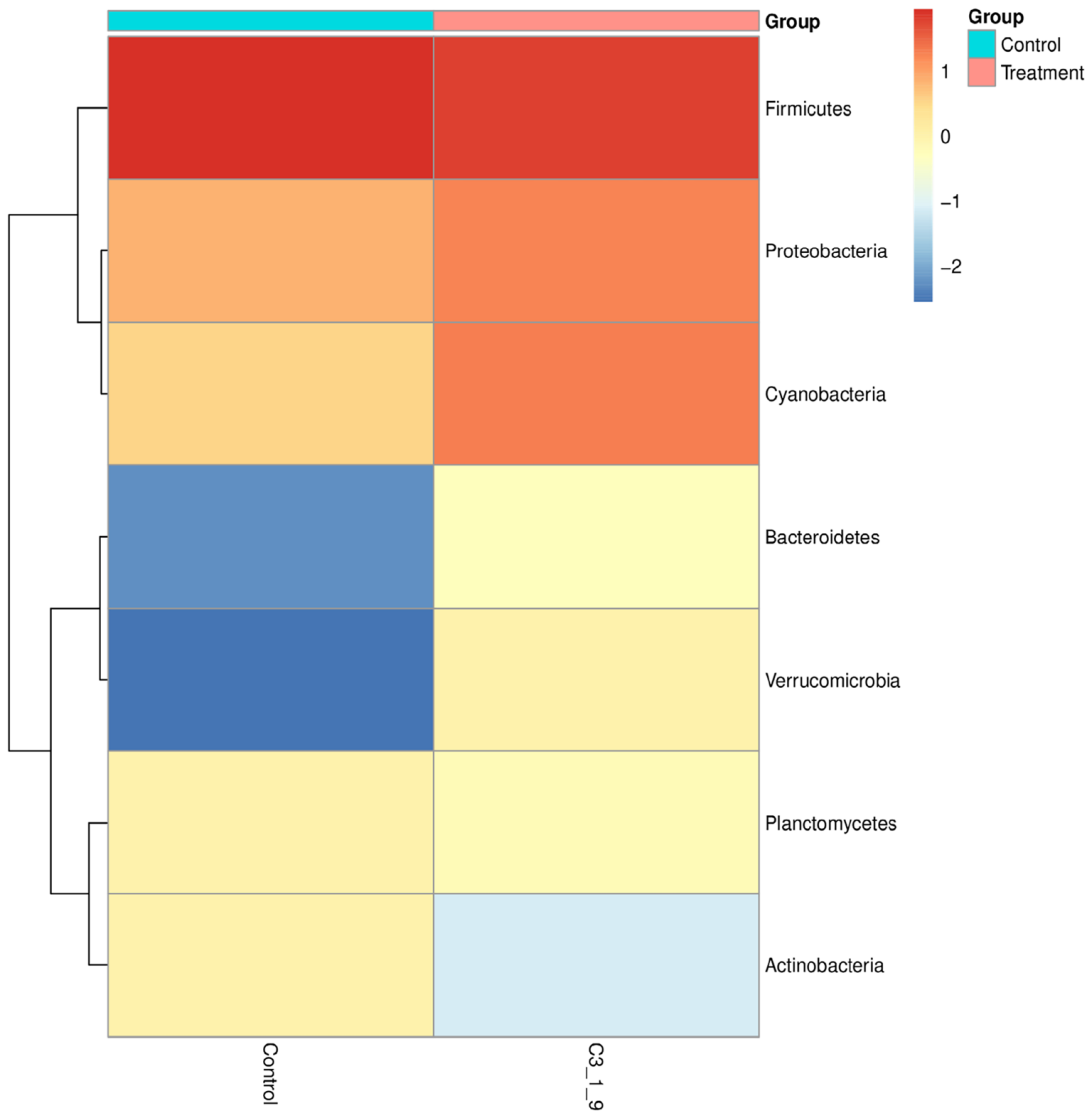
The heat map analysis of the vanilla microbiota composition at the genus level for *Bacillus velezensis* ZN-S10 fermented samples (C3-1-9) as compared with the control group.

According to the heat map analysis (Fig. 6), the vanilla microbiome at the genus level presented significant differences in the relative abundance across the bacteria-fermented samples (C3-1-9) and the control groups. Hence, an increase of *Firmicutes, Proteobacteria,* and *Cyanobacteria* was observed while *Bacteroidetes*, *Verrucomicrobia*, and *Planctomycetes* were reduced with a significant decrease of *Actinobacteria* on the microbiome of bacteria-treated vanilla samples. Similarly, *Firmicutes* were remarkably increased in the control group, and *Proteobacteria and Cyanobacteria* were reduced while significant decreases were observed in *Bacteroidetes* and *Verrucomicrobia*, with a slight increase in *Planctomycetes* and *Actinobacteria* in contrast with the microbiome of the treated vanilla. Carbajal-Valenzuela et al.^27^ stated that vanilla plants' root and stem system can be accumulated with *Actinobacteria*, which can later be inhabited in post-harvested pods. Similarly, Ling et al.^67^ studied root-grafted watermelon showed a high dominance of *Firmicutes*, *Actinobacteria*, and *Cyanobacteria*, *Proteobacteria* rather than noon-grafted watermelon. This suggests that the soil's vanilla microbial community might accumulate more in roots, but mostly important to the abundance of bacteria in the pods that survive during the blanching process. However, these findings require further research.

**Figure 7:**
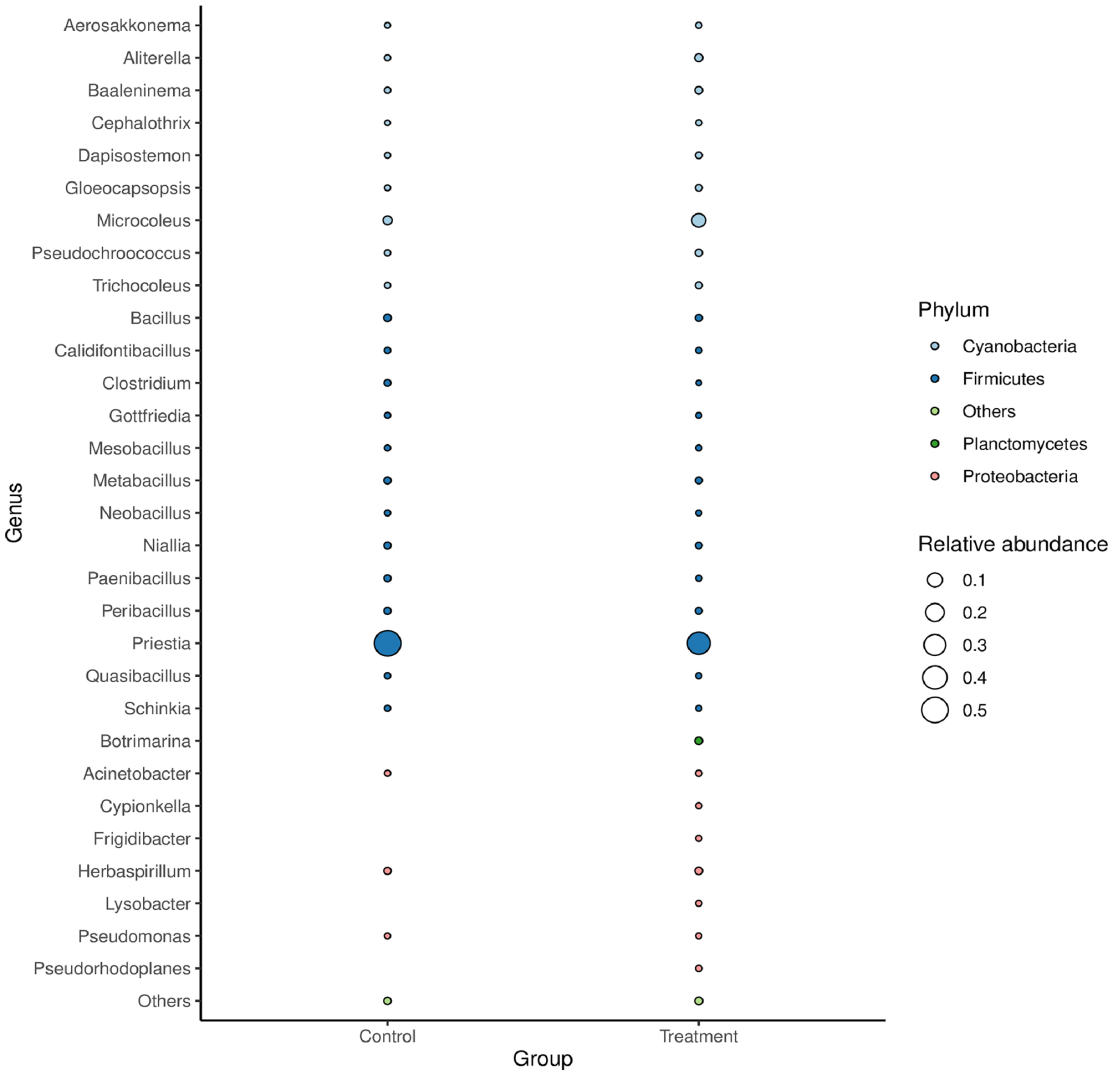
The taxonomic bubble plot of the bacteria in vanilla at the relative abundance at genus and phylum levels for the control and treatment groups. Circle sizes represent the relative abundance, and colors indicate the bacterial phylum groups.

Figure 7 shows the bubble plot had four bacterial classes that include; *Firmicutes, Cyanobacteria, Planctomycetes,* and *Proteobacteria* among others were the most abundant in the microbiome of naturally fermented vanilla and samples fermented with *B. velezensis*. Hence, at the genus level, *Priesta* had a higher relative abundance of 0.5 for *Firmicutes* phylum on both control and treated vanilla bean samples. Studies have shown that the more abundant of Firmicutes with Actinobacteria in the bacterial phyla is commonly due to the production of secondary metabolites such as volatile compounds^68,69^. Therefore, the fermentation of vanilla with the most abundance of the bacteria phyla exhibited that volatile compounds were produced after the *B. velezensis* ZN-S10 inoculation on the pods. The results, owing to the bacteria phyla reported suggest that bacteria play a role in altering the aroma and flavor-volatile compounds in *V. planifolia*.

**Figure 8:**
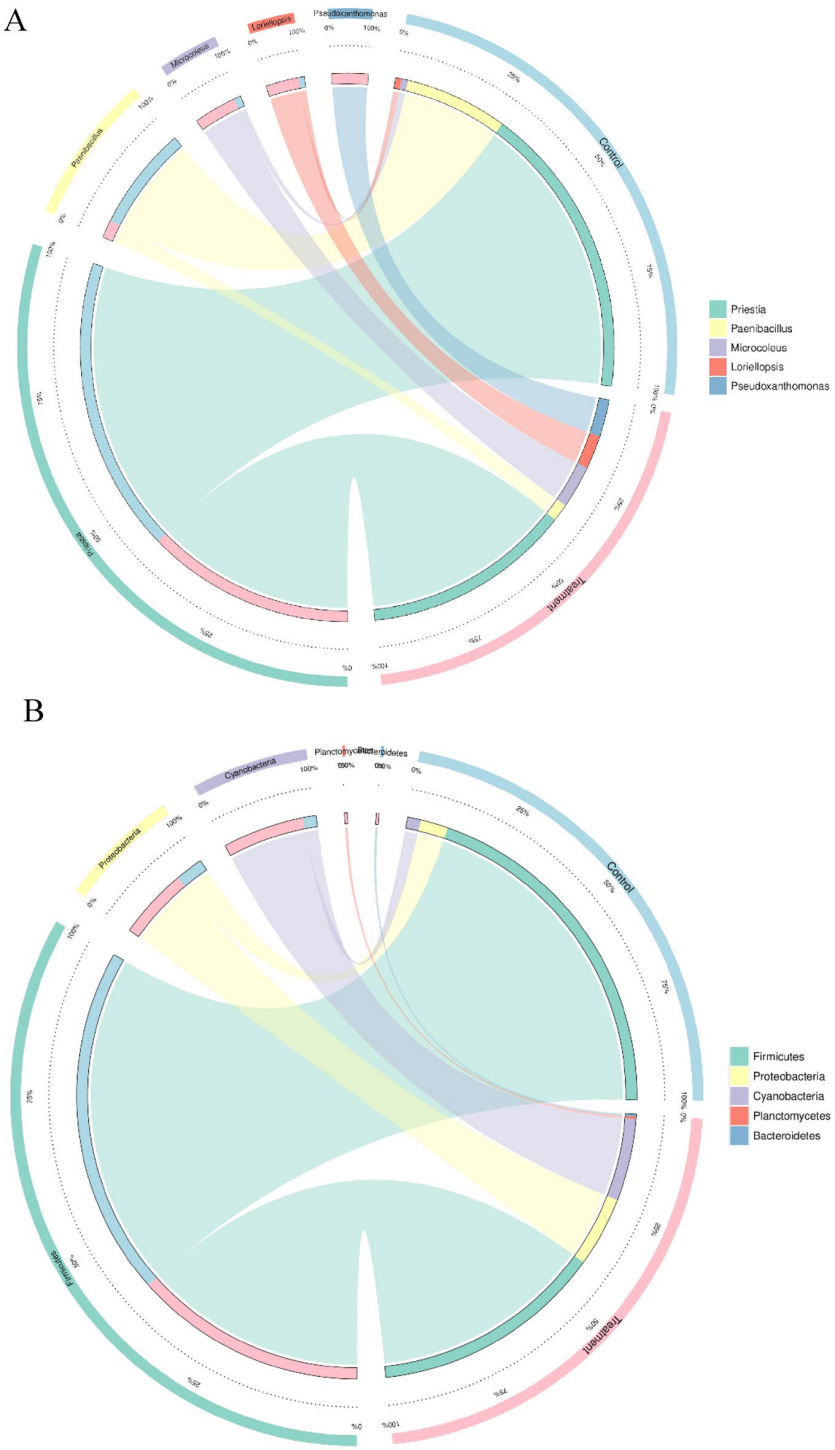
Circos representation of the most abundant bacterial genera between naturally fermented vanilla (control) and *B. velezensis* ZN-S10 fermented samples. (**A**) The circus plot of the relative abundance (%) at the phylum level. (**B**) The circos plot at the genus level of the percentage of relative abundance percentages (%) for the microbiota found in vanilla beans coated with *B. velezensis* ZN-S10 and non-coated vanilla pods (control).

The distinct bacterial communities were presented in a circos plot at genus and phylum levels (Fig. 8) that was detected in naturally fermented vanilla (control) and the vanilla treated with *B. velezensis* for fermentation purposes during the curing process. We identified 5 different genera in both the control and treated vanilla samples. Hence, at the genus level, the treated vanilla samples had dominant *Priesta* (45%)*,* while the naturally fermented vanilla had 55% dominance. Interestingly, at this level larger fractions of *Pseudoxanthomonas* (96%)*, Microcoleus* (90%)*, Loriellopsis* (95%)*,* and *Paenibacillus* (97%)*,* as compared to the control group (Fig. 8A). Nevertheless, the control vanilla microbiota was slightly supplemented with *Microcoleus* (10%)*,* and *Loriellopsis* (5%); also showing a lower abundance of *Paenibacillus* (3%), *Psedoxanthomans* (4%), which was contrary to the microbiota of the treated vanilla. Therefore, the *Priesta* or *Bacillus* were presented as the predominant bacterial community at the genus level. It should also be noted that the *Firmicutes* were the most dominant phylum (Fig. 8B) as observed with higher relative abundance at the phylum level. Li et al.^70^ found that pepper plants in allyl isothiocyanate fumigated soil had an increased relative abundance of *Pseudoxanthomonas* with a reduced abundance of *Planctomycetes* and *Acinetobacter* at genus level. The changes in the bacteria communities were similar to the significant differences between *B. velezensis*-treated vanilla and the control. The reports of these studies also exhibited that soil treatment should be monitored for soil bacteria and other microorganism communities. Thus, the variation of the bacteria genera in the vanilla beans might be affected by soil conditions. Moreover, studies with disease-resistant tobacco cultivar HB202 compared with a susceptible XY3 cultivar showed that the HB202 variety had higher bacteria communities in contrast to the XY3 variety^70^. Differences in vanilla bean varieties might support different microbial-network communities, especially relative abundances of bacteria genera after the curing process of vanilla.

## Conclusions

In this research, *Bacillus velezensis* ZN-S10 was successfully isolated from *Vanilla planifolia* and was used for bacterial fermentation in green-matured non-cured vanilla beans. The bacteria communities were also analyzed with the NGS technique, where 16S rRNA gene sequencing to explore the genus and phylum level of the bacteria population involved. The annotations provided by the sequence data, multiple aspects of the development and biochemistry of vanilla beans could be understood to be involved in the colonization of non-cured vanilla beans, while microorganisms are important in fermentation for the curing process in *V. planifolia*. The 16S rRNA gene sequencing in this study was shown to be very crucial for the understanding of the bacterial ecology, abundance, and relationship taxonomy to improve the fermentation procedures done for the curing process to achieve high-quality cured vanilla beans. The findings of the bacterial communities suggested that at the genus level *Priesta* were dominant; hence were involved in the colonization or fermentation of *V. planifolia*. Moreover, at the phylum level, the Bray–Curtis distance revealed a higher relative abundance of *Firmicutes*, hence these bacteria had an important role in the curing process of the vanilla beans. Further research on the cured vanilla beans cannot only be used to extract the bacteria responsible for fermentation but for natural vanilla extract as well as for the preparation of other vanilla products. This article proved that the isolated bacteria from the vanilla, *B. velezensis* ZN-S10 could control the bacterial communities during the fermentation process for the manufacturing of safe vanilla food products (Supplementary Information).

### Supplementary Information

Supplementary Information.
