# Supplementary material for: The isolation and identification of Bacillus velezensis ZN-S10 from vanilla (V. planifolia), and the microbial distribution after the curing process
Source: Sci Rep. 2024 Jul 16;14:16339. doi: 10.1038/s41598-024-66753-z (PMC11252412; doi:10.1038/s41598-024-66753-z)
Supplement: Supplementary file 1 — Supplementary Information. [file 41598_2024_66753_MOESM1_ESM.docx]

SUPPLEMENTARY FILE

NCBI details on the Accession number for Bacillus velezensis strain ZN-S10 isolated from Vanilla planifolia used during the construction of the phylogenetic tree:

**Website link:**

[**https://www.ncbi.nlm.nih.gov/nucleotide/CP102933.1?report=genbank&log$=nucltop&blast_rank=1&RID=PSM1SUJP016**](https://www.ncbi.nlm.nih.gov/nucleotide/CP102933.1?report=genbank&log$=nucltop&blast_rank=1&RID=PSM1SUJP016)

Bacillus velezensis strain ZN-S10 chromosome, complete genome

Sequence ID: **CP102933.1**

Length: 3929792

Number of Matches: 9

Range 1: 96969 to 98456
